# Radial Expansion of the Nephrogenic Zone in the Fetal Human Kidney During Advanced Pregnancy: A Microanatomical Look at a Little Noticed Process

**DOI:** 10.1155/ijne/7571982

**Published:** 2025-03-20

**Authors:** Will W. Minuth

**Affiliations:** Institute of Anatomy, University of Regensburg, Regensburg D-93053, Germany

**Keywords:** fetal human kidney, impairment of nephrogenesis, nephrogenic compartment, nephrogenic zone, nephron development, radial expansion

## Abstract

**Introduction:** The experiences with preterm and low birth weight babies indicate a special vulnerability of their kidneys, since different kinds of noxae can evoke the termination of nephron formation. This leads to oligonephropathy, which is associated with serious consequences for health in the later stages of life. While the clinical aspects have been intensely investigated, only few pathological data point to the initial traces left by the noxae. Up to this date, only the reduction in the width of the nephrogenic zone (NZ) and the lack of here occurring basophilic S-shaped bodies were reported.

**Methods and Materials:** The relationship between the arising nephron and its structural neighbors changes throughout the developmental progress. Locally, this determines the vertical width of the NZ reflected by the radial expansion of both the parenchyma and the interstitium. Since information about the origin, the site, and the involved structures is not available, the related microanatomical features were recorded.

**Results:** The data reveal that the renal vesicles, comma-shaped bodies, and S-shaped bodies are unequally distributed in the NZ. Due to their progressive sizes, it has an influence on the local vertical width of the NZ. This parameter is registered as the distance between the inner side of the renal capsule and the proximal pole of the respective stage of the nephron anlage. The vertical width can be further subdivided: the constant height of the district of progenitor cell recruitment and the variable height of the area of nephron shaping. Exclusively here, the radial expansion of the shaping nephron stages can be noticed. It starts at the section border between the head and the conus of the related collecting duct ampulla by positioning the primitive renal vesicle. While the respective proximal pole stays mounted next to the connecting tubule of a previously developed nephron, the distal pole sticks between the head and the conus at the CD ampulla for linking the future connecting tubule. This causes that henceforth the medial aspect of the extending renal vesicle, comma-shaped body, or S-shaped body stages radially expands in close proximity to the elongating conus of the CD ampulla.

**Conclusion:** Between the arising nephron stages and the elongating conus of the CD ampulla, a linked radial expansion occurs. This new finding is essential to identify the extent of targeting of noxae that subsequently leads to a reduction in the width of the NZ.

## 1. Introduction

Both phylo- and ontogenetic events directly affect the development of the human kidney. It starts with the formation of the pronephros at 4 weeks of gestation to continue with the mesonephros during Week 5 [1]. Surprisingly, only its distal part develops further to the lasting metanephros. This is a composed organ, which is built up by 6–8 renal lobes. The first glomeruli become visible during the ninth week. By 36 weeks of gestation, the formation of new nephrons is terminated. The endowment refers to the total number of nephrons, with which an individual is born [2]. It is the product resulting from the personal renal genetic blueprint, the in utero situation, the effect of environmental exposures, and the running cell and molecular biological processes [3].

During the development of the fetal human kidney, the process of nephrogenesis is not constant—it accelerates over time. More than half of the total number of nephrons develops in the last 3 months of pregnancy [4]. Studies of the glomeruli population revealed that the average number of formed nephrons in an adult human kidney is approximately 1 million, and there can be variations. Depending on the internal and external conditions, the number of glomeruli can range from as low as 200,000 to as high as 2.7 million [5, 6]. In this context, it was recognized that a low nephron endowment increases susceptibility to renal stress and chronic kidney disease [7].

The clinical experiences with preterm and low birth weight babies indicate that their kidneys are especially vulnerable in the last months of pregnancy [8]. Different kinds of noxae such as malnutrition, diabetes, vitamin deficiency of the mother, insufficiency of the placenta, and hyperoxia and also drugs can evoke the termination of nephrogenesis [9]. This leads to oligonephropathy with severe consequences for health in later life [10, 11].

The numerous data collected in the clinical environment for preterm and low birth weight babies are in strong contrast to the limited literature on dealing with the cellular and molecular targets of the listed noxae. For example, only few pathological findings point to the initial damage in the outer cortex of the fetal human kidney. For the externally situated nephrogenic zone (NZ), the loss of basophilic S-shaped bodies was reported [12]. In addition, it was shown that in gestational controls, the vertical width of the NZ is not more than 150 μm, while in the case of preterm babies it is significantly smaller with a width of 100 μm [13]. However, details regarding the related structural changes were not reported. For the subjacent maturation zone, a reduced number and the occurrence of atypical glomeruli, which exhibit an extended Bowman's space and a shrunken glomerular tuft, were described [14].

As the microanatomical base for the fetal human kidney during advanced pregnancy is incomplete, only limited data have been reported up to date. The general features of the evolving glomeruli of the fetal human kidney from mid- to late gestation were described previously [15]. However, only recently the morphological peculiarities of the NZ [16, 17], the shaping of the nephron [18], the mutual patterning with its structural neighbors [19], and the different parts of the local interstitium [20] have been communicated. It is essential to conduct the ultrastructural analysis of the NZ via modern transmission and scanning electron microscopic techniques. Another reason for the scarce literature might be that investigations dealing with the impairment of nephrogenesis have been primarily made by using animal models. Unfortunately, a much-needed verification of the generated results for the fetal human kidney could not be found in the related literature. All this is of special importance, since current cell and molecular biological data indicate that not only shared but also divergent features in the animal and fetal human kidneys determine the development of a nephron [21–23].

The development of a nephron in the fetal human kidney during advanced pregnancy is a chain of multiple links. At the start, it is based on the recruiting of the progenitor cells [22], and then, it is the subject of the successively running process of nephron shaping [18]. It is mirrored by the arise of the transient stages of nephron anlage such as the mesenchymal-to-epithelial transition (MET), the different renal vesicle stages, and the comma- and S-shaped bodies [3, 24]. It is typical that the development of these transient stages is restricted to the NZ [17]. While they increase in size, changing shapes have also been observed. In parallel, numerous interactions occur between the respective structural neighbors such as the collecting duct (CD) ampulla or the local interstitium including the perforating radiate artery. This indicates that the development of a nephron is not a separate operation but an interactive process. An essential part of the development is the installation of the shaping nephron [25]. It coincides with a radial expansion of the parenchyma and the interstitium. An unanswered question is how far it determines the vertical width of the NZ in the fetal human kidney during advanced pregnancy. Due to the lack of reports on the concrete site of nephron extension, the measurement points, the metric dimensions, and the possible interactions with the structural neighbors, the microanatomical analysis shown in this work was conducted. Consequently, this work and the findings within provide the first basic insights, which will support a reliable pathological assessment and a definite interpretation of data generated by current cell and molecular biological methods.

## 2. Material and Methods

### 2.1. Kidney Preparation

For obtaining a morphological look at the NZ and the here occurring radial expansion of the forming nephron, one must focus on the precise positions of the transient stages of nephron anlage such as the nephrogenic niche, pretubular aggregate, MET, renal vesicle, and comma- and S-shaped body [17]. In the fetal human kidney during advanced pregnancy, the NZ lines as a thin strip along the inner side of the renal capsule. Consequently, damage to this region during the histological preparation must be prevented. Therefore, a fetal human kidney is held only on its hilum so that the touching of the renal capsule with fine forceps is avoided. At least 10 kidneys were analyzed.

### 2.2. Histological Section Plane

For the microscopic analysis of the histological sections, comparable perspectives are required. To realize it, a fixed kidney is cut from the renal capsule toward the papilla of a lobe. Following this advice, the section plane lines along the axis of the vertically running CD tubules and at the same time perpendicular to the renal capsule.

### 2.3. Selection of Specimens

For the here shown illustrations, specimens of the fetal human kidneys of gestational age between Weeks 16, 18, and 25 were selected from the stock of preparations used for the Course of Microscopic Anatomy for Medical Students at the University of Regensburg in Germany. These stages during kidney development are of special interest, since during the last months of pregnancy most nephrons are formed in the outer cortex of the expanding organ [4]. The size of these organ stages is in such a dimension that each kidney can be embedded as a whole for histology so that the touching and excising of the outer cortex is avoided.

### 2.4. Sample Preparation

The tissue blocks were fixed in paraformaldehyde solution and embedded in paraffin wax. Then, sections of 5-μm thickness were cut and stained with hematoxylin–eosin solution for analysis by an optical microscope. The screening of the stained sections was performed by a Leica DM750 microscope (Leica Microsystems, Wetzlar, Germany). The single shaping stages of nephron anlage were analyzed with a HI Plan 63x/0.75 objective lens. Images were taken with a Basler Microscopy Pulse 5.0 camera (Basler AG, Ahrensburg, Germany). The screened specimens had a normal microanatomical look and were of good histological preservation. Causes of death, congenital abnormalities, renal cysts, or other pathological alterations were not visible.

### 2.5. Screening of Specimens

More than 3000 images were available, which had been analyzed for earlier performed investigations dealing with the shaping (Minuth 2020) [18], the mutual patterning of the nephron with its structural neighbors [19], and the interjacent interstitium [20]. From this stock, first from each of the analytical groups, 5 representative images were selected, which illustrate the basic stations of the radial nephron expansion and/or which show the relationship between the transient stages of nephron anlage and the CD ampulla. Thereby, the section plane lines along the vertically orientated CD ampulla and perpendicular to the renal capsule. Then, one of these typical images was selected for the here shown illustrations. For obtaining information dealing with the metric parameters in the microscopic images and for inserting the necessary labels, the program CorelDRAW 2021 (Corel Corporation, Munich, Germany) was used. It is important to point out that the here presented metric parameters are first orientation values. Further morphometric data from healthy kidney stages as a standard must be generated, when intact with pathological specimens are compared.

### 2.6. Interpretation of Images

To visualize the morphological changes while the radial expansion of the shaping nephron, original images are presented. These depict that the proximal pole (fix point, lower side of the image) of the currently developing stage of nephron anlage rests next to the connecting tubule of a previously developed nephron. Since the space is limited at this site, the distal pole (mobile point, upper side of the image) of the currently developing stage of nephron anlage shifts radially in concert with the elongating conus of the CD ampulla during the proceeding development. Thereby, the entire overlying structures such as the tip and head of the CD ampulla, the pool of progenitor cells, the pretubular aggregate, and the covering renal capsule are correspondingly translocated as a parcel. Thereby, its composition is not disturbed.

## 3. Results

### 3.1. Unequal Distribution of Nephron Stages Causes a Heterogeneous Width

**Figure 1:**
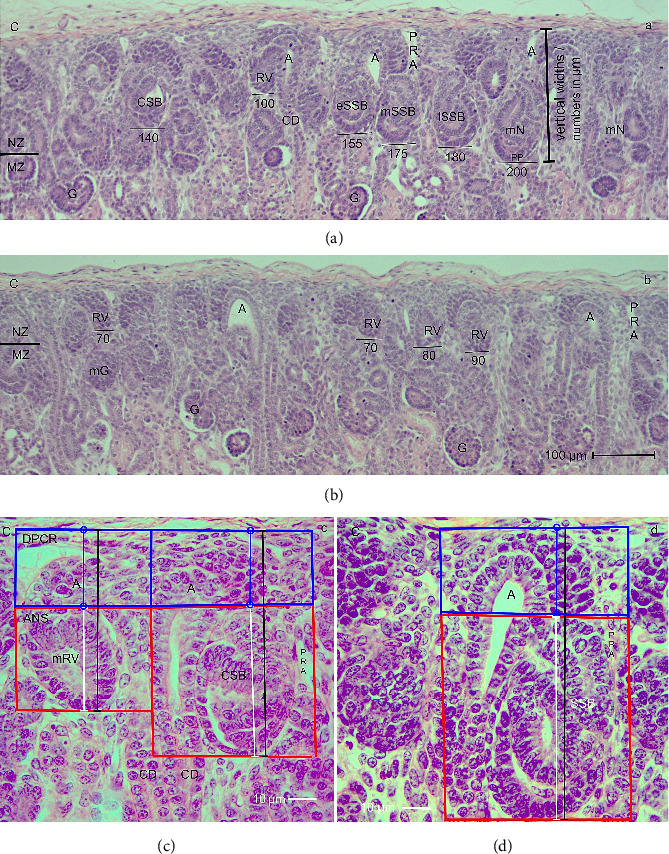
(a–d) Look at the nephrogenic zone (NZ) in the fetal human kidney during advanced pregnancy by the optical microscope. It lines as a thin strip between the inner side of the renal capsule (C) and the maturation zone (MZ). The NZ is heterogeneously composed, since differently far developed and consequently progressively sized transient stages of nephron anlage are contained. (a) The histological section demonstrates the presence of renal vesicle (RV) stages, comma-shaped bodies (CSB), early (e), mid (m), and late (l) S-shaped bodies (SSB), and the start of a maturing nephron (mN). (b) In contrast, other sections of the same kidney show predominantly renal vesicles. This indicates that the width of the nephrogenic zone (black vertical bar), which is measured from the proximal pole (PP) of the respective stage of nephron anlage to the inner side of the renal capsule (black numbers), is not constant but differs. (c and d) Each of the transient stages of nephron anlage develops in a nephrogenic compartment. The height (black vertical bar) reflects the current vertical width of the nephrogenic zone at this site. Most important is that it can be subdivided into the height of the district of progenitor cell recruitment (DPCR, blue vertical bar in the blue frame) and the height of the subjacent area of nephron shaping (ANS, white vertical bar in the red frame). Solely here, either (c) a mature renal vesicle (mRV), a comma-shaped body (CSB), or (d) an S-shaped body (SSB) is contained. Due to their special position, one can see that exclusively the vertical height of the ANS increases, while the renal vesicle, comma- and S-shaped body stages develop. In contrast, the height of the DPCR is not affected. CD: collecting duct tubule, A: collecting duct ampulla, PRA: perforating radiate artery, G: glomerulus, mG: maturing glomerulus.

In the fetal human kidney during advanced pregnancy, the formation of new nephrons is restricted to the NZ (Figures 1(a) and 1(b)). It is recognized as a thin strip of developing parenchyma and interstitium. The external side of the NZ contacts the inner side of the renal capsule, while its bottom side faces the maturation zone. Surprisingly, the screening of the NZ by the optical microscope reveals that the stages of nephron anlage are heterogeneously distributed. When sections from the same kidney are compared, one recognizes that in some areas, only few renal vesicles but numerous comma- or S-shaped bodies are present (Figure 1(a)). In contrast, in other areas, predominantly renal vesicle stages but few comma- and S-shaped bodies are recognized (Figure 1(b)). As a first result, the occurrence of differently far developed nephron stages indicates that the developmental progress in the NZ is not paralleled but individually controlled. The second result is that due to the unequal distribution of the differently sized nephron stages, the vertical width of the NZ (Figures 1(a) and 1(b)) varies from area to area.

### 3.2. Structural Borders in the NZ

Each of the nephron stages in the NZ can be allocated to a nephrogenic compartment. These are aligned in one row and next to each other (Figures 1(c) and 1(d)). In each of these compartments, either a renal vesicle, comma-shaped (Figure 1(c)), or an S-shaped body (Figure 1(d)) is contained. At the top, a nephrogenic compartment is covered by the inner side of the renal capsule. The lateral border is defined by a vertically orientated CD ampulla. The contralateral border is limited by a perforating radiate artery, which ascends from the maturation zone through the NZ for joining the rete capsulare in the renal capsule. In the space between the lateral borders, the respective stage of nephron anlage develops. The base of a nephrogenic compartment is represented by the transversely orientated connecting tubule of a previously developed nephron.

By drawing a transverse double line along the section border between the head and the conus of the CD ampulla and the connecting tubule of a previously developed nephron, a nephrogenic compartment can be subdivided (Figures 1(c) and 1(d)). The upper part was named the district of progenitor cell recruitment (DPCR). At the top, it contacts the renal capsule. Underneath, the pool of the stromal/interstitial cells and the nephrogenic progenitor cells is visible. Thereby, the competent nephrogenic progenitor cells face the tip of the related CD ampulla. Somewhat sideways and opposite the head of the CD ampulla, the pretubular aggregate is positioned. At its proximal end, the MET takes place. Underneath the transverse double line, the area of nephron shaping (ANS) is present. Here, either a renal vesicle stage, comma-shaped body, or S-shaped body is visible. The respective medial aspect of the different nephron stages faces the conus of the CD ampulla.

After screening the nephrogenic compartments in the NZ using the optical microscope, it was deducted that the vertical height of the DPCR remains constant. While the renal vesicle up to the comma-shaped body develops, the typical example depicts that its vertical height is around 30 μm (Figure 1(c)). It only increases slightly during the development of the S-shaped body and the successive MET (Figure 1(d)). In contrast, the respective height of the ANS changes. Due to the progressively sized stages of nephron anlage, it is still small, when a mature renal vesicle is present (Figure 1(c)). However, it increases while the formation of the comma-shaped body takes place. The height increased to its maximum, when an S-shaped body is developing (Figure 1(d)). This result indicates that the vertical width of the NZ, which is measured from the inner side of the renal capsule to the proximal pole of the respective stage of nephron anlage, depends on the local developmental progress. Since the formation of nephrons in the NZ is not paralleled, it varies from one nephrogenic compartment to the next. Nevertheless, similarly far progressed stages can occur side by side.

### 3.3. Guide Rail for Nephron Formation

**Figure 2:**
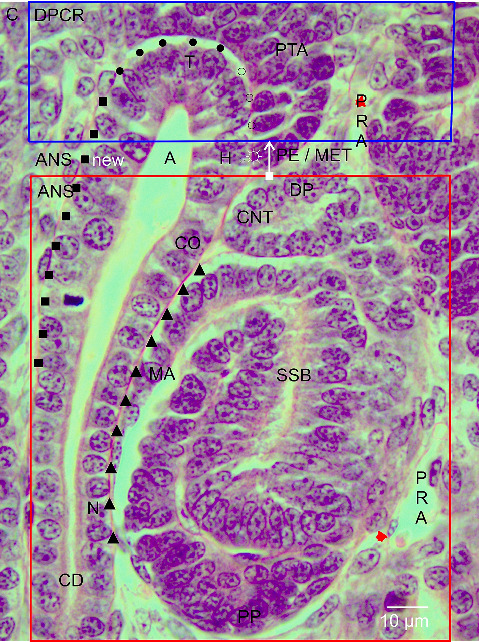
Look at the starting nephron anlage in the nephrogenic zone of the fetal human kidney during advanced pregnancy by the optical microscope. This arises at the proximal end (PE) of the pretubular aggregate (PTA), which is framed by the distal pole (DP) of the previously formed S-shaped body (SSB) and the head (H) of the facing collecting duct (CD) ampulla (A). The white vertical arrow indicates that during the running mesenchymal-to-epithelial transition (MET) between the proximal end of the pretubular aggregate located in the district of progenitor cell recruitment (DPCR) and the connecting tubule (CNT) of the previously formed S-shaped body located in the subjacent area of nephron shaping (ANS), a new shaping area (ANS new) is opening. Remarkable are the different interstitial spaces: Black spots indicate the clear interface between the nephrogenic progenitor cells and the tip (T) of the CD ampulla. Light circles point to the clear interface between the medial side of the pretubular aggregate and the head of the CD ampulla. The light circle with spines indicates the adhesion between the future primitive renal vesicle and the head of the ampulla. At this site, the new connecting tubule will form. Black squares show the faint interstitium at the free conus (CO) of the CD ampulla. At the contralateral side, the black triangles point to the narrow interstitial gap between the medial aspect (MA) of the S-shaped body and the facing conus of the CD ampulla. C renal capsule, CO conus and N neck of CD ampulla, PP proximal pole, PRA perforating radiate artery.

The formation of a nephron is not a separate process but occurs by a close interaction with the facing and vertically orientated CD ampulla. It starts in the small space between the inner side of the renal capsule and the here ending tip of a CD ampulla (distance of about 20 μm). Here, the tunica muscularis of the renal capsule faces the interstitial/stromal progenitor cells. Underneath the stromal cells, one to two layers of the nephrogenic progenitor cells occur. The innermost layer is positioned next to the basal aspect of the epithelial progenitor cells, which are contained in the tip of the CD ampulla (Figure 2). This peculiar site represents the nephrogenic niche. Strikingly, the bodies of the nephrogenic cells and the epithelial progenitor cells do not touch but are separated by a clear interface.

When a vertically orientated CD ampulla is screened by the optical microscope, it can be observed that it is built up by a monolayered and polarized epithelium and that it is structured by sectors (Figure 2). The blindly ending sector near the renal capsule is represented by the tip. Somewhat sideward, its head is localized. This is faced by the medial side of the tear drop-like pretubular aggregate. It is evident that a clear interface is present not only at the nephrogenic niche but also at the medial side of the pretubular aggregate. A deeper analysis is required to determine whether it is the same. At the transverse section border between the head and the conus of the CD ampulla, the connecting tubule of a currently developing renal vesicle stage, a comma-shaped body, or an S-shaped body is linked. The respective medial aspect of the shaping nephron faces the conus of the CD ampulla.

The interstitial sites, which surround a CD ampulla, are special (Figure 2). As mentioned, a clear interface is visible at the tip and head. This is terminated, when the medial part of the proximal end at the pretubular aggregate contacts the transverse section border between the head and the conus of the CD ampulla. Especially interesting is that between the medial aspect of the extending renal vesicle stages, the comma-shaped body or the S-shaped body, and the conus at the CD ampulla, a surprisingly close and even congruent structural relation exists. Later, it is replaced by a narrow and vertically ascending interstitial cleft. The situation at the contralateral surface of the CD ampulla is completely different. At the section border between the head and the conus of the CD ampulla, the clear interface ends. It is replaced by a faint interstitium, which lines along its conus to the neck.

### 3.4. Lasting Position of the Pretubular Aggregate

While the competent nephrogenic progenitor cells are induced by a series of morphogenic proteins in the nephrogenic niche, the imprinted progenitor cells translocate from the tip sideward to the head of the related CD ampulla for assimilation in the pretubular aggregate (Figure 2). Its thin distal end points to the renal capsule. It remains connected with the nephrogenic progenitor cells, which are located next to the tip of the related CD ampulla. In contrast, the broad proximal end of the pretubular aggregate stays positioned next to the connecting tubule of a previously developed nephron.

The constant position of the proximal end at the pretubular aggregate is basic for the initial formation of a nephron (Figure 2). The lateral part of its proximal end is exposed to the perivascular interstitium of a vertically lining perforating radiate artery. The middle of its proximal end is mounted next to the connecting tubule of a previously developed nephron. Equally important, the medial part of its proximal end adheres at the section border between the head and the conus of the related CD ampulla.

### 3.5. Site of Nephron Installation

**Figure 3:**
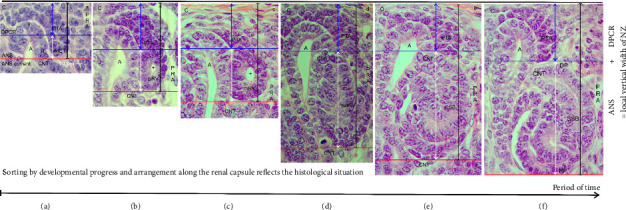
(a–f) Look at the vertical width (black vertical bar) of the nephrogenic zone in the fetal human kidney during advanced pregnancy by the optical microscope. In the composite image, the stages of nephron anlage are sorted by the developmental progress. The leveling is made along the renal capsule as it is observed in the histological section. The vertical width is determined by measuring the distance between the inner side of the renal capsule and the proximal pole (PP, fix point) of the respective nephron stage. One can see that the vertical width depends on the currently available stages of nephron anlage. (a) The example shows that during the mesenchymal-to-epithelial transition (MET), it is 52 μm. (b) When the primitive renal vesicle (pRV) develops, the vertical width rises to 65 μm. (c) While the mature renal vesicle (mRV) forms, the vertical width reaches 77 μm. (d) When the extended renal vesicle (extRV) arises, it exhibits 115 μm. (e) While the comma-shaped body (CSB) appears, the vertical width is 127 μm. (f) During the development of the S-shaped body (SSB), the vertical width reaches a maximum of 160 μm (f). The illustration further points out that changes in the vertical width of the nephrogenic zone are restricted to the area of nephron shaping (white double arrow). In the overlying district of progenitor cell recruitment, comparable changes are not observed. Red line: base of the area of nephron shaping (ANS), blue line: bottom of the district of progenitor cell recruitment (DPCR), blue double arrow: height of the district of progenitor cell recruitment, white double arrow: height of the area of nephron shaping, C: renal capsule, A: collecting duct ampulla, PTA: pretubular aggregate, CNT: connecting tubule, PRA: perforating radiate artery, +: primary lumen.

The next topological link in the chain of nephron formation is the MET (Figures 2 and 3(a)). It is restricted to the proximal end of the pretubular aggregate. Here, the primitive renal vesicle arises. It is first recognized as an open epithelial clamp at the proximal end of the pretubular aggregate (Figure 2). Later, it closes to a circle (Figure 3(a)). Yet, the primitive renal vesicle is covered by a single-layered polarized epithelium and a small lumen is visible in its center. The basal lamina at its outer side faces the evolving interstitium.

During the next developmental steps, the lateral part of the distal pole at the primitive renal vesicle remains connected with the overlying pretubular aggregate (Figure 3(a)). It is exposed to the perivascular interstitium of a here ascending perforating radiate artery. In contrast, the medial part of the distal pole shows a separation. Then, at the medial aspect of the primitive renal vesicle, the initial adhesion at the section border between the head and the conus of the CD ampulla is replaced by a real attachment to enable the invasion of the future connecting tubule.

### 3.6. Claim of Expansion

During the screening of the NZ by the optical microscope, one recognizes that differently far advanced stages of nephron anlage are contained (Figures 1(c) and 1(d)). Thereby, each of them is part of a nephrogenic compartment. When the stages of nephron anlage are sorted first according to the developmental progress and then aligned along the renal capsule, the specimens demonstrate that the shaping activity of the forming nephron is restricted to a space solely intended for this purpose (Figure 3). While the MET proceeds at the proximal end of the pretubular aggregate, it is still part of the DPCR in a nephrogenic compartment (Figures 2 and 3(a)). However, the backdrop is changing, when the primitive renal vesicle separates (Figure 3(b)). The yet arising mature (Figure 3(c)) and extended (Figure 3(d)) renal vesicle stages, the comma-shaped body (Figure 3(e)), and the S-shaped body (Figure 3(f)) develop henceforth exclusively in the newly opened ANS.

With the help of a composite image, it is shown that the shaping of the nephron correlates with a vertical expansion of the nephrogenic compartment (Figure 3). When the height is measured between the inner side of the renal capsule and the proximal pole of the respective stage of nephron anlage, the vertical width can be recorded. In the demonstrated example, it is 52 μm for the MET (Figure 3(a)), 65 μm for the primitive (Figure 3(b)), 77 μm for the mature (Figure 3(c)), 115 μm for the extended (Figure 3(d)) renal vesicle stage, and 127 μm for the comma-shaped body (Figure 3(e)), and 160 μm for the S-shaped body (Figure 3(f)). Furthermore, the structural changes are restricted to the ANS. In this way, the renal capsule remains at the same level, while the proximal pole of the respective nephron stage appears to be dislocated to the bottom. The reason for this “false” impression is that we compare nephron stages of quite different ages and of different local origins. For example, the demonstrated S-shaped body (Figure 3(f)) was initiated much earlier, and it was forming at a quite different site than the primitive renal vesicle (Figure 3(b)). This again depicts that the younger the nephron stage, the closer its fix point (proximal pole) is positioned at the transverse section border lining between the head and the conus of the CD ampulla. In this context, it is especially important to generate the numeric distribution of the single nephron stages in the NZ of the intact kidney by a morphometric program and to then compare it with an impaired kidney.

### 3.7. Parameters of Radial Expansion

**Figure 4:**
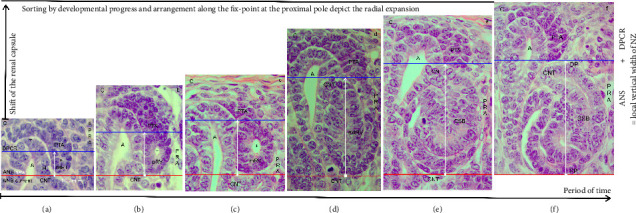
(a–f) Look at the radial expansion (white vertical arrow) of the stages of nephron anlage in the nephrogenic zone of the fetal human kidney during advanced pregnancy by the optical microscope. In contrast to Figure 3, the leveling of the nephron stages in the composite image is made along the common base point at the proximal pole. The radial expansion is determined by measuring the distance between the proximal pole (PP, fix-point) and the connecting tubule (CNT) at the distal pole (mobile point) of the respective nephron stage. (a) The example shows that during the mesenchymal-to-epithelial transition (MET), it is 20 μm. (b) When the primitive renal vesicle (pRV) develops, it rises to 32 μm. (c) While the mature renal vesicle (mRV) forms, it reaches 45 μm. (d) When the extended renal vesicle (extRV) arises, it exhibits 80 μm. (e) While the comma-shaped body (CSB) appears, it is 93 μm. (f) During the development of the S-shaped body (SSB), the radial expansion reaches a maximum of 110 μm. One can recognize that the radial expansion exclusively determines the local vertical width of the nephrogenic zone. Thereby, the structural changes are restricted to the area of nephron shaping of the nephrogenic compartment. Red line: base of the area of nephron shaping (ANS), blue line: bottom of the district of progenitor cell recruitment (DPCR), C: renal capsule, A: collecting duct ampulla, PTA: pretubular aggregate, PRA: perforating radiate artery, +: primary lumen.

A closer look at the vertical width of the NZ revealed that it varies, that it depends on the currently available stages of nephron anlage, and that these are restricted to the ANS (Figure 3). To obtain further insights into the radial expansion, the stages of nephron anlage already shown in Figure 3 were sorted by the developmental progress. Then, they were aligned not along the renal capsule, but along the base of their structural origin (Figure 4). In the corresponding composite image, the proximal pole of the shaping stages of nephron anlage reflects the respective fix point, which is next to the connecting tubule of a previously formed nephron. The respective measurement point is the distal pole at the connecting tubule of the currently shaping stage of nephron anlage. Hence, the distance between these two points reflects the radial expansion of the shaping nephron.

In the demonstrated specimen, an initial vertical distance of around 10 μm is measured between the connecting tubule of the previously formed nephron and the adhesion site of the pretubular aggregate at the head of the CD ampulla (Figure 2). In addition, the shown example indicates that during the MET, the radial expansion is 20 μm (Figure 4(a)). When the primitive renal vesicle develops, it increases to 32 μm (Figure 4(b)). While the mature renal vesicle forms, the radial expansion reaches 45 μm (Figure 4(c)). When the extended renal vesicle arises, it extends to 80 μm (Figure 4(d)). Then, when the comma-shaped body appears, the radial expansion increases to 93 μm (Figure 4(e)). During the development of the S-shaped body, the radial expansion reaches a maximum of 110 μm (Figure 4(f)). When this kind of illustrated image sorting would be performed by an appropriate morphometric program, one could obtain precise information on whether the radial expansion of the shaping nephron is statistically different between an intact and a harmed kidney.

### 3.8. Common Radial Expansion

**Figure 5:**
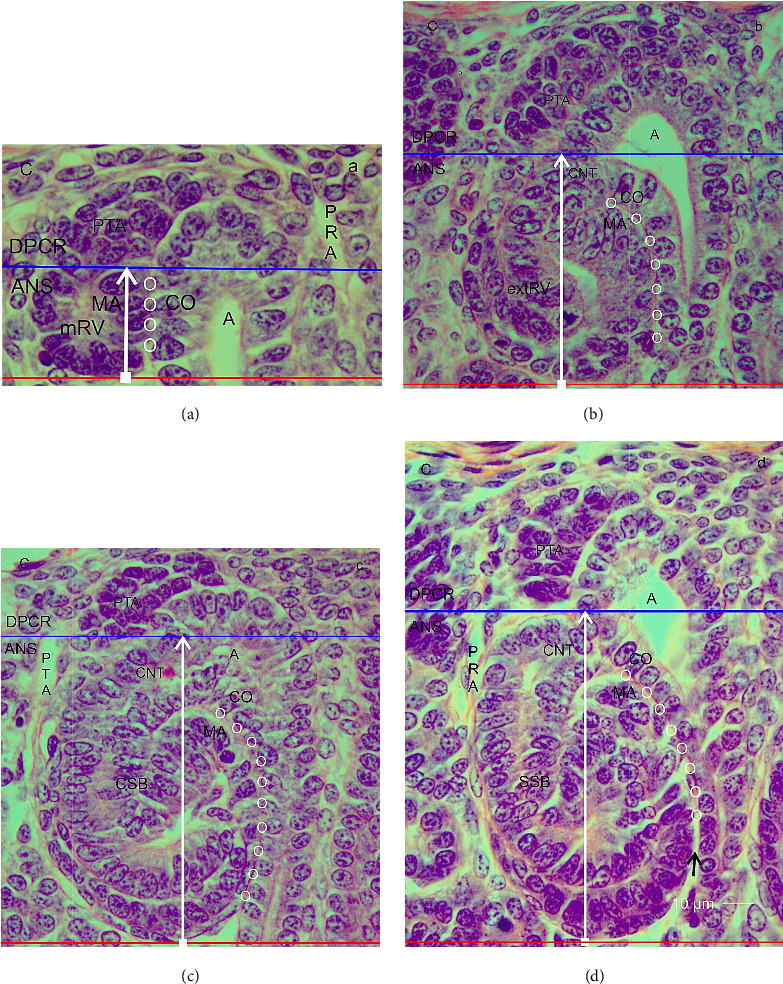
(a–d) View at the common radial expansion of the transient stages of nephron anlage and the conus of the collecting duct (CD) ampulla (A) in the nephrogenic zone of the fetal human kidney during advanced pregnancy by the optical microscope. While the radial expansion (white vertical arrow) occurs, as well as between the medial aspect (MA) of (a) the mature renal vesicle (mRV), (b) the extended renal vesicle (extRV), (c) the comma-shaped body (CSB), and (d) the S-shaped body (SSB) and the conus (CO) of the related CD ampulla, a surprising closeness and congruency (white circles) is registered. During the further development, the closeness is disbanded and replaced by a narrow interstitial cleft (short black arrow). Red line: base of the area of nephron shaping (ANS), blue line: bottom of the district of progenitor cell recruitment (DPCR), C: renal capsule, CNT: connecting tubule, PRA: perforating radiate artery.

The increase in the vertical width of the NZ caused by the radial expansion is driven not by one, but by a myriad of shaping nephrons (Figures 3 and 4). However, it is based on a less noticed structural cooperation (Figure 5). While the mature (Figure 5(a)) and extended (Figure 5(b)) renal vesicles are forming, the demonstrated specimens depict that the respective future connecting tubule invades at the section border between the head and the conus of the related CD ampulla. This structural connection causes that during the radial expansion, the medial aspect of the shaping nephron elongates together with the conus of the CD ampulla. In addition, between the medial aspect of the renal vesicle stages (Figures 5(a) and 5(b)), the comma-shaped bodies (Figure 5(c)), the early S-shaped bodies (Figure 5(d)), and the conus of the related CD ampulla, a surprising closeness and congruency is registered. While the S-shaped body matures, it is replaced by a narrow interstitial cleft (Figure 5(d)). It is obvious that during the radial expansion of the shaping nephron, there must also be a corresponding elongation of the facing perforating radiate artery.

### 3.9. From One Nephron Anlage to the Next

**Figure 6:**
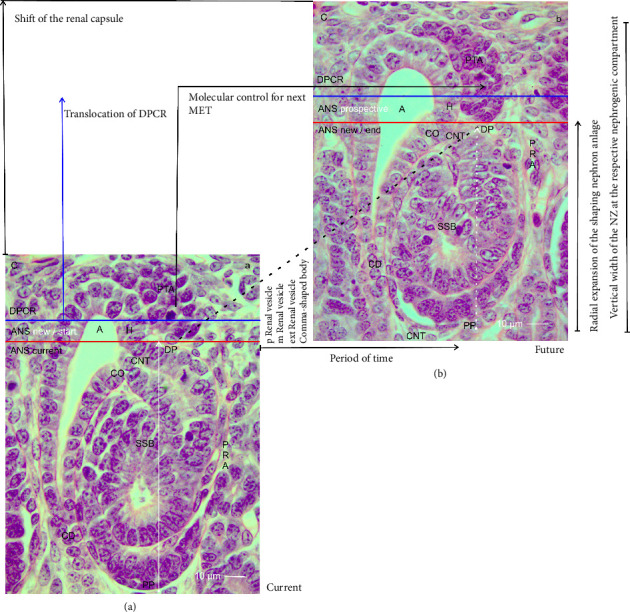
(a–b) The composite image shows the putative radial expansion from one nephron anlage to the next in the nephrogenic zone of the fetal human kidney during advanced pregnancy by the optical microscope. (a) On the left side, one must imagine that the currently forming S-shaped body (SSB) will develop after its radial expansion (white vertical arrow) into a maturing nephron. In the mid of the composite image, the prospective but successive development from a primitive, mature, extended renal vesicle to a comma-shaped body is indicated. (b) On the right side of the composite image, the position of the future S-shaped body in the next nephrogenic compartment of the nephrogenic zone is outlined. While the future radial expansion (dotted white vertical arrow) within the area of nephron shaping (ANS) occurs, the distal pole (DP) of the future S-shaped body distances itself from the proximal pole (PP). This causes a translocation of the overlying district of progenitor cell recruitment (DPCR) including a shift of the renal capsule (C). Strikingly, the radial expansion of the shaping nephron exclusively takes place in concert with the elongating conus (CO) of the CD ampulla (A). The dashed black line shall indicate that the formation can happen not only in the demonstrated section plain but also punctually and in circumference around the head of the ampulla. Further the period of time is unknown, in which the primitive renal vesicle develops up to the S-shaped body. Unclear is also, by which molecular signals and how often a primitive renal vesicle is formed via the mesenchymal-to-epithelial transition at the proximal end of the pretubular aggregate (PTA). Red line: base of the area of nephron shaping, blue line: bottom of the district of progenitor cell recruitment, CD: collecting duct, H: head, CO: conus, A: collecting duct ampulla, CNT: connecting tubule, PRA: perforating radiate artery.

The radial expansion of the nephron anlage demonstrates that not only its shaping but also the necessary initiation of the next nephron generation is an indispensable further link. To illuminate it, a composite image was produced (Figure 6). On the left-hand side, a nephrogenic compartment including an S-shaped body is shown (Figure 6(a)). This will remain to become a maturing nephron in the subjacent maturation zone. At the top of the S-shaped body, an active MET at the proximal end of the pretubular aggregate can be observed. In the middle of the composite image, the radially expanding renal vesicle stages up to the comma-shaped body are indicated. On the right-hand side, the position of the resulting S-shaped body is shown (Figure 6(b)).

A look at the composite image indicates a series of less considered parameters, which determine the development from one nephron anlage to the next (Figure 6). When the transverse border at the head of the CD ampulla is elongated to the proximal pole of the currently shaping nephron, an abscissa becomes visible. It points to the unknown duration of time, in which the development from the MET up to the S-shaped body occurs. The crossing ordinate indicates the state of the radial expansion. It is minimal, when an early stage of nephron anlage such as a primitive renal vesicle within the ANS is present. It becomes maximal when the S-shaped body arises. In the case that the earlier mentioned noxae interfere with the process of expansion, a reduction in the width of the NZ at those sites can be registered by a sophisticated morphometric program.

The composite image further indicates that the radial expansion in the NZ starts in a gusset, which is framed by the head of the CD ampulla, the related connecting tubule of a previously formed nephron, and the proximal end of the respective pretubular aggregate (Figures 2 and 6(a)). The dashed diagonal line indicates the base point of an expandable space (Figure 6(a)), in which the new nephron anlage will start to shape (Figure 6(b)). In the case that noxae harm this site of origin, the consequence is that the next nephron will not form, since a continuing link in the chain is broken. The specimen further depicts that exclusively at the proximal end of the pretubular aggregate, the MET becomes active. Obviously, this can only take place if the S-shaped body as the last transient stage of nephron anlage is evolving. In other words, when noxae aim at the earlier stages of the nephron anlage or when they interfere with the shaping process of the S-shaped body, the next generation of nephrons will most probably not form.

Finally, the specimens point out that during the shaping of the nephron, the tip and head of the CD ampulla, the overlying progenitor cells contained in the cap condensate, the pretubular aggregate, the subcapsular interstitium, and the renal capsule are lifted as a parcel (Figure 6). This happens without altering the structural configuration in the overlying DPCR.

## 4. Discussion

### 4.1. Asynchronous Formation of Nephrons

Typical for the fetal human kidney is that more than half of the nephrons arise in the last 3 months of pregnancy [4]. In the clinical environment, the progress of developmental activity is registered as organ growth. For example, between the gestation Weeks 32 and 38, the length of the fetal kidney increases from 3.6 to 4.2 cm and its width from 2 to 2.2 cm so that the volume enlarges from 14.5 to 21.6 cm^3^ [26, 27]. The actual expansion takes place in the outer cortex. It includes the local fetal parenchyma and the related interstitium [20]. In this case, the formation of new nephrons, which starts with the arise of the transient stages of nephron anlagen such as the renal vesicles, comma-shaped bodies, and S-shaped bodies, is restricted to the externally situated NZ (Figures 1(a) and 1(b)) [16, 17]. The subsequent maturing of the developing nephron occurs in the subjacent maturation zone.

It was shown in previous works that the NZ extends as a strip with a vertical width of 150 μm underneath the renal capsule [13, 15]. When screened using an optical microscope, one can see that the transient stages of nephron anlage occur exclusively here (Figures 1(a) and 1(b)). Each of these transient stages develops in a nephrogenic compartment (Figures 1(c) and 1(d)). By comparing the nephrogenic compartments, one will recognize that in each of them, a differently far developed and consequently differently sized stage of nephron anlage is contained. The generated illustrations also show that in some of the nephrogenic compartments, a mature renal vesicle (Figure 1(c)) is found, while in others either an extending renal vesicle, a comma-shaped body (Figure 1(c)), or an S-shaped body (Figure 1(d)) is contained. Due to the heterogeneous distribution, one can conclude that the appearance of the stages of nephron anlage in the NZ is not synchronized. Instead, it is time-delayed and controlled on the base of an individual nephrogenic compartment.

### 4.2. Unequal Vertical Width of the NZ

It was reported that in gestational controls, the vertical width of the NZ is not more than 150 μm, while in the group of preterm babies it is significantly smaller at a width of 100 μm [13]. However, the current data depict that the vertical width of the NZ in an intact kidney varies from one nephrogenic compartment to the next (Figures 1(c), 1(d), 3). Thereby, the height of its externally situated DPCR stays constant, while the height of its subjacent ANS differs. Since only here either a renal vesicle stage, a comma-shaped body, or an S-shaped body is present, this alone reflects the true vertical width of the NZ at a particular point (Figure 3). Thus, a region in the NZ with a dominating number of primitive or mature renal vesicles (Figure 1(c)) will indicate only a minimal vertical width. In contrast, a neighboring region containing either extended renal vesicles, comma-shaped bodies, or S-shaped bodies (Figure 1(d)) in the nephrogenic compartments will show a correspondingly larger vertical width.

### 4.3. Radial Expansion and Its Main Players

The radial expansion of the shaping nephron starts with the MET at the proximal end of the pretubular aggregate (Figures 2, 3(a), 4(a)). As a result, the primitive renal vesicle is established (Figures 3(b), 4(b)). While its proximal pole is mounted next to the connecting tubule of a previously formed nephron, the lateral part of the distal pole at the primitive renal vesicle remains for the moment connected with the overlying pretubular aggregate. However, the medial part of its distal pole shows a separation for the subsequent adherence at the section border between the head and the conus of the CD ampulla. For the developing mice kidney, it was shown that the cell adhesion molecule cadherin-6 plays an essential role in this process [28]. However, for the fetal human kidney, comparable data are not available.

The initial adhesion alters to a real attachment for linking the future connecting tubule at the distal pole of the currently forming primitive renal vesicle with the upper conus of the CD ampulla. The resulting connection causes that the distal pole as the mobile point of the shaping nephron is radially translocated in concert with the elongating conus of the CD ampulla (Figure 5). The microscopic images further depict that between both structures, a previously unexplored congruency and proximity exists. It can be imagined that the cooperation between the conus of the CD ampulla and the medial aspect of the shaping nephron is a preferred target of the noxae. A harming of this striking constellation might be the cause for the earlier described reduction in the width of the NZ [13, 29].

### 4.4. At the Border Between Stemness and Shaping

The structural base of a newly forming nephron in the fetal human kidney during advanced pregnancy is positioned at the proximal end of the pretubular aggregate (Figure 2). Thereby, it faces the transverse section border between the head and the conus of the related CD ampulla. At the same time, it marks the transverse border between the overlying DPCR and the underlying ANS (Figures 1(b) and 1(c)). At this peculiar border, as well as the MET, the generation of the primitive renal vesicle as its partial separation occurs. Consequently, these structural key points force us to question whether the development of a renal vesicle at the proximal end of the pretubular aggregate is only a single event or whether a repetitive release is possible [30]. Furthermore, it is unclear whether these key points are damaged by the mentioned noxae. They may aim at the partial separation of the primitive renal vesicle from the pretubular aggregate as well as at the simultaneous adhesion of its distal pole to the CD ampulla [28, 31–34]. It is obvious that a disturbance of these key points by the mentioned noxae would block the start of the shaping of a future nephron generation (Figure 6).

### 4.5. What Is Visible Versus What Is Going On

The present investigation depicts that the radial expansion is restricted to the ANS (Figures 1(c), 1(d), 3). Thereby, it determines the local vertical width of the NZ. It is little when a primitive renal vesicle arises, and it reaches a maximum during the development of an S-shaped body. Comparable alterations in the overlaying DPCR are not observed. For obtaining further insights into the expansion, the stages of nephron anlage were sorted according to their size for the alignment along their common structural base (Figure 4). The composite image equalizes the time-delayed developmental start, and it enables to depict the eccentric but radial expansion of the nephron stages. When both perspectives are compared, it reminds us that there is a difference in what we perceive in a histological section (Figure 3) and what is going on in the NZ (Figure 4). A peculiar meaning in this context has the common expansion of the conus at the CD ampulla and the medial aspect at the shaping nephron (Figure 5). This again directs the search for initial traces left by the noxae to less considered targets, for example, a disturbance of the molecular texture at the proximal pole of the primitive renal vesicle during its mounting. In addition, there could be interference at the section border between the head and the conus of the CD ampulla while the adherence of the primitive renal vesicle occurs, and a faulty separation of its distal pole from the pretubular aggregate takes place or a harming begins while the shaping nephron extends in the radial direction.

Finally, the generated data point to the less noticed molecular control, by which a renal vesicle starts to shape, while the underlying S-shaped body is changing its development to become a maturing nephron (Figure 6). It appears that there are at least two different programs. The first program controls the shaping activity from the MET up to S-shaped body (Figure 6(a)). The other pilots the developmental activity between the connecting tubule of the currently shaping S-shaped body and the proximal end in the overlying pretubular aggregate. Consequently, it must be taken into account that the mentioned noxae may interfere not only with the developmental activity of the shaping nephron but also with the consecutive release of the renal vesicle at the pretubular aggregate (Figure 6(b)). It is therefore possible that during the separation of the primitive renal vesicle, for example, an evoked standstill of the cell motility at the proximal end of the pretubular aggregate occurs. In turn, this would lead to a complete lack of the extending renal vesicle, comma-shaped body, and S-shaped body stages. The most critical issue is that the corresponding literature on this topic is not available in the related databases.

## 5. Conclusions and Recommendations

The present investigation illuminates microanatomical parameters of the NZ in the fetal human kidney. First, it was noticed that a uniform vertical width of the NZ does not exist. Instead, the vertical width depends on the locally available transient stages of nephron anlage. Second, the radial expansion, which determines the vertical width, does not take place in the DPCR, but it is restricted to the ANS of a nephrogenic compartment. Third, the radial expansion starts at the section border between the head and the conus of the CD ampulla. Fourth, the radial expansion is active during development of the renal vesicle, comma-, and S-shaped body stages. Fifth, the radial expansion takes place in concert with the elongating conus of the CD ampulla. Hence, the data generated in this work enable the analysis to reveal how far the identified structures are affected by noxae impairing nephrogenesis.

## Data Availability

The datasets generated and/or analyzed during the current study are not public available due to the ongoing research but are available from the corresponding author upon reasonable request.
